# Intraoperative thermal infrared imaging in neurosurgery: machine learning approaches for advanced segmentation of tumors

**DOI:** 10.1007/s13246-023-01222-x

**Published:** 2023-01-30

**Authors:** Daniela Cardone, Gianluca Trevisi, David Perpetuini, Chiara Filippini, Arcangelo Merla, Annunziato Mangiola

**Affiliations:** 1 Department of Engineering and Geology, University G. d’Annunzio Chieti-Pescara, Pescara, Italy; 2 Department of Neuroscience, Imaging and Clinical Sciences, University G. d’Annunzio Chieti-Pescara, Chieti, Italy

**Keywords:** Thermal infrared imaging, Neurosurgery, Brain tumor segmentation, Machine learning, Classification

## Abstract

Surgical resection is one of the most relevant practices in neurosurgery. Finding the correct surgical extent of the tumor is a key question and so far several techniques have been employed to assist the neurosurgeon in preserving the maximum amount of healthy tissue. Some of these methods are invasive for patients, not always allowing high precision in the detection of the tumor area. The aim of this study is to overcome these limitations, developing machine learning based models, relying on features obtained from a contactless and non-invasive technique, the thermal infrared (IR) imaging. The thermal IR videos of thirteen patients with heterogeneous tumors were recorded in the intraoperative context. Time (TD)- and frequency (FD)-domain features were extracted and fed different machine learning models. Models relying on FD features have proven to be the best solutions for the optimal detection of the tumor area (Average Accuracy = 90.45%; Average Sensitivity = 84.64%; Average Specificity = 93,74%). The obtained results highlight the possibility to accurately detect the tumor lesion boundary with a completely non-invasive, contactless, and portable technology, revealing thermal IR imaging as a very promising tool for the neurosurgeon.

## Introduction

Surgical resection plays a central role in the management of brain tumors. The extent of resection is one of the most important predictors of patient outcome, together with the patient’s age and performance status, tumor histology, and molecular markers [1].

The extent of tumor resection affects the patient’s survival, quality of life, and the possible evolution time towards higher-grade neoplastic forms. However, especially in cases of tumors with infiltrative features like gliomas, the actual border of resection between tumor and healthy tissue can be sometimes hard to detect with standard microneurosurgical techniques. Therefore, some residual tumor tissue may be involuntary left in place, thus negatively influencing oncological results. Moreover, brain does not allow an indiscriminate supramarginal resection of the tumor since patients may develop major neurological deficits.

To ensure an adequate extent of resection, several intraoperative techniques have been introduced.

The more commonly used are neuronavigation, intraoperative ultrasound (iUS), 5-aminolevulinic acid (5-ALA) fluorescence, and intraoperative magnetic resonance (iMR). All these methods have some strengths and limitations. Neuronavigation is widely available and easy to be interpreted, but relies on preoperative MR (which cannot be updated during the resection) and is limited by brain shift [2]. iUS is a cheap and effective on-line technique that can be boosted by some technological advances as neuronavigation and contrast-enhancement (CEUS), but is severely operator-dependent and limited by residual tissue volume, surgery induced artifacts and previous treatments in cases of recurrent tumors [3]. 5-ALA fluorescence is also widely used and very effective to improve extent of resection in high grade gliomas (HGGs) [4], with lower accuracy in other tumor types [5] and some limitations in recurrent HGGs cases [6]. iMR is the less diffused technology and is limited by high costs, need for dedicated operative room spaces and equipment, and long interruptions of surgical work flow [7].

The difficulty given by the infiltrative nature of some types of tumors and the increasing need to use non-invasive imaging techniques in intraoperative contexts made thermography an ideal candidate for the development of a new approach.

In the present study, intraoperative thermal infrared imaging (iIRI) has been used to assess tumor boundaries by means of a machine learning-based approach. IRI is based on a passive, non-contact assessment of the temperature pattern of the object of measurement relying on a thermal camera device. Relevant literature works are reported in the Literature Review section.

In the present work, different machine-learning based models have been compared relying on time and frequency domain input features, relative to thermal IRI of brains. The originality of the work consists mainly in relying on thermal spectral features, which have proven to be more sensitive to detecting tumors from healthy tissue. The developed approach reveals the capability of intraoperative thermal IRI to accurately detect the cancer lesion boundary with the aim to develop an integrative tool for conservative purposes in neurosurgery.

### Literature review

The research field on image segmentation for diagnostic purposes is broad and there is a huge variety of scientific works on new methodologies, ranging from liver [8, 9], to breast [10] and to vertebrae [11] segmentation.

With reference to the use of thermal IRI, there is a consistent literature in the biomedical field, especially for diagnostic purposes. Several studies have been performed to detect breast cancer [12–15] and skin tumors, i.e. melanoma [16–18], whereas other studies investigated the capacity of the technique to classify different kind of diseases related to macro- or micro-circulatory impairment, i.e. Varicocele [19, 20] or Raynaud Phenomenon [21, 22].

Although the literature about the application of thermal IRI in neurosurgery is sparse, it is known that the presence of brain neoplasms alters the thermal homeostasis of the surrounding tissue. Indeed, studies on animal and human models reported a lower temperature profile of primary tumors of glial origin than the surrounding parenchyma [23–25]. Gorbach et al. showed that glial tumors have a temperature 0.5–2.0 °C lower than the surrounding healthy brain parenchyma [24]. Numerous factors can determine the decrease in cerebral flow and / or metabolic activity and induce a decrease in the temperature of the lesion. Factors responsible for decreased local brain flow in primary brain neoplasms include low density of neoplastic microcirculation, peritumor edema, poor metabolism of the cortex overlying the neoplastic lesion, and tumor necrosis. Reduced cerebral blood flow is characteristic of both primary and metastatic brain tumors, although the latter have, in most cases, a hyperthermic profile. Brain neoplasm has also been shown to induce a “disconnection effect” such that cortical gray matter metabolism is reduced in the area overlying the tumor [26].

Differently from tumors of glial origin, brain metastases are hyperthermic, as reported by Gorbach et al. [24] and Kateb et al. [27]. The latter, in a clinical case of intracortical metastases from melanoma in a 76-year-old woman, documented a clear thermal demarcation between metastases (36.4 °C) and healthy brain parenchyma (33.1 °C) as revealed by intraoperative measurements from a thermal imaging camera [27]. The biological heterogeneity of the neoplasms, however, influences the temperature pattern among the different lesions [24].

More recently Kastek et al. confirmed the possibility to use iIRI and observed an altered temperature pattern of the cancer area with respect to the healthy parenchyma. For instance, in a patient with a cyst due to a metastatic tumor, they reported a decrease of 2.6 °C in the surface of the cyst compared to the surrounding tissue [28].

Sadeghi-Goughari et al. performed intraoperative thermal imaging coupled with artificial tactile sensing and artificial neural network to develop a method for the diagnosis and localization of brain tumors and to estimate geometrical and thermal properties of the detected tumor. The procedure was validated on a patient with a parafalcine meningioma and thermal parameters extracted from thermal IRI process were utilized to train the proposed neural network to estimate tumor temperature and depth. The method reached an error equal to 0.0627 °C and 0.7015 mm, for thermal property and depth respectively [29].

## Materials and methods

### Participants

Thirteen patients (8 males; age range (61.46 ± 8.28) years old), diagnosed with a neoplastic brain lesion and eligible for surgical resection, were recruited in the Neurosurgery Unit of the Santo Spirito Hospital in Pescara, Italy.

The need for surgical intervention was established independently by using conventional clinical indications and surgery was performed blindly from iIRI recordings. Informed consent was obtained from all the patients, who were selected from a cohort of cases enrolled according to the protocol approved by the Local Ethic Committee (protocol number 08/21.05.2020).

Table 1 resumes the information about location, volume and specific category of the tumors. Average thermal values and standard deviation of tumor and healthy tissues are reported in the Table 1 relatively to the baseline phase.

**Table 1:** Demographic and tumor information of the patients. Basal temperature of tumor and healthy area

Case	Age	Gender	Tumor Location	Tumor Side	Pathology	Tumor Volume (cm^3^)	Tumor distance from cortical surface (cm)	Basal temperature (mean ± standard deviation) (°C)	
								Tumor area	Healthy area
1	73	M	T–O	R	Glioblastoma	56.20	0	34.09 ± 1.12	33.62 ± 1.95
2	51	F	T	L	Meningioma	2.00	0.6	33.32 ± 0.50	34.16 ± 0.78
3	68	M	F	L	Glioblastoma	54.75	0	34.62 ± 0.86	33.73 ± 0.74
4	63	M	F post-Cing-CC	R	Oligodendroglioma Grade III	32.80	0.6	29.06 ± 1.36	29.76 ± 1.37
5	67	F	F–P	L	Metastasis (Kidney carcinoma)	6.30	1.5	35.03 ± 0.47	35.09 ± 0.66
6	52	M	F	L	Glioblastoma	1.25	0.6	36.72 ± 0.66	35.30 ± 1.22
7	56	M	F	R	Astrocitoma Grade II-III	34.50	0.3	32.39 ± 1.26	31.38 ± 2.07
8	62	M	F	L	Glioblastoma	64.50	1.5	32.46 ± 0.83	32.70 ± 1.06
9	66	F	P	L	Glioblastoma	21.60	0.3	32.87 ± 0.80	32.92 ± 0.93
10	52	F	F–T	R	Glioblastoma	41.00	1.5	34.16 ± 0.64	33.95 ± 1.37
11	49	M	F	L	Glioblastoma	60.00	0	34.17 ± 0.57	33.15 ± 1.09
12	75	M	T	R	Metastasis (SCLC)	100.00	0	34.61 ± 0.68	34.52 ± 0.68
13	65	F	P	L	Glioblastoma	32.60	0	31.07 ± 0.58	31.89 ± 1.05

*M* male, *F* female, *T* temporal lobe, *O* occipital lobe, *F* frontal lobe, *Cing* cingulate gyrus, *CC* corpus callosum, *P* parietal lobe, *R* right hemisphere, *L* left hemisphere, *SCLC* small cell lung cancer

### Procedure and data acquisition

During neurosurgery, a thermal infrared camera was used to assess the superficial temperature of the cortex. Specifically, a FLIR SC660 (FLIR, Wilsonville, OR, USA) (640 × 480 bolometer FPA, sensitivity/noise equivalent temperature difference: < 30 mK @ 30 °C, field of view: 24° × 18°) was employed. The camera focused the exposed brain region at a distance of about 60 cm. Concurrently to the thermal imaging acquisition, the visible imaging of the exposed region was acquired by Logitech C920 HD PRO camera, to segment the tumor region relying on the co-registration between visible and thermal imaging.

**Fig. 1:**
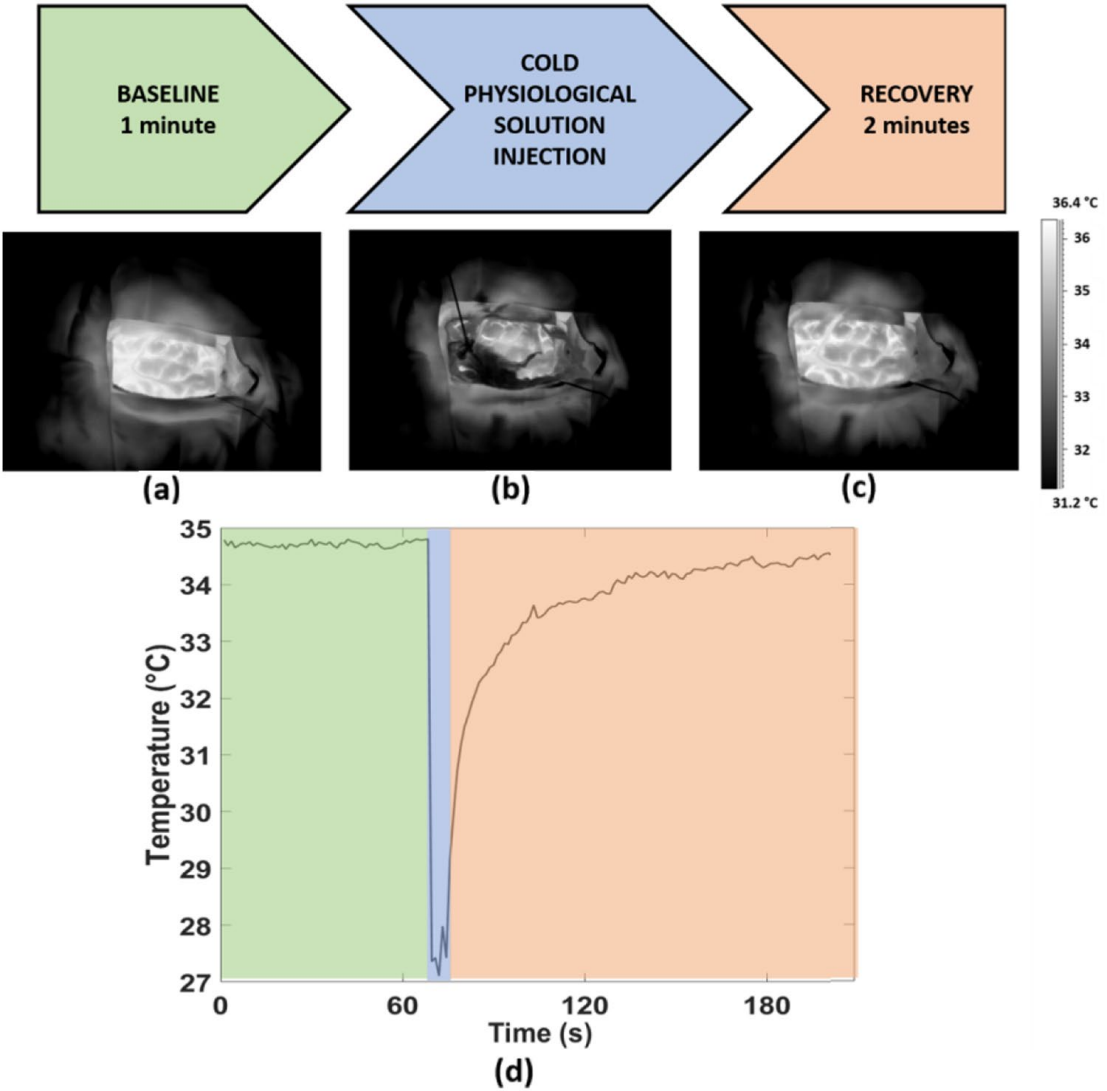
Experimental procedure consisting in a baseline (BL) phase (highlighed in green), cold physiological solution injection (highligheted in light blue) and recovery (REC) phase (highligheted in orange); (**a**),(**b**),(**c**) Thermal IR images of the exposed brain tissue relative to BL, injection and REC phases respectively; d) thermal signal of one representive pixel over time

The experimental procedure is described in Fig. 1. Firstly, one minute of baseline (BL) was considered to measure the baseline temperature of the cortex (Fig. 1a). Subsequently, a cold physiological solution (at a temperature of 10 °C) was injected to provide a cold stress to the cortex (Fig. 1b), and finally, two minutes of recovery (REC) were contemplated to investigate the different thermal behavior of the healthy and tumor tissue (Fig. 1c). Thermal imaging was acquired at a frame rate of 5 Hz (i.e. 5 frames per second). Figure 1d shows the thermal signal of one random pixel of the exposed cortex.

During the whole experimental procedure, the environmental conditions were kept stable (i.e. temperature: 22 °C, humidity: 50–60%).

### Tumor segmentation and optical co-registration

Tumor boundary were defined by the neurosurgeon on the visible image of the exposed cortex on the basis of the projection of the tumor area on the brain surface, relying on the MRI of the patient.

**Fig. 2:**
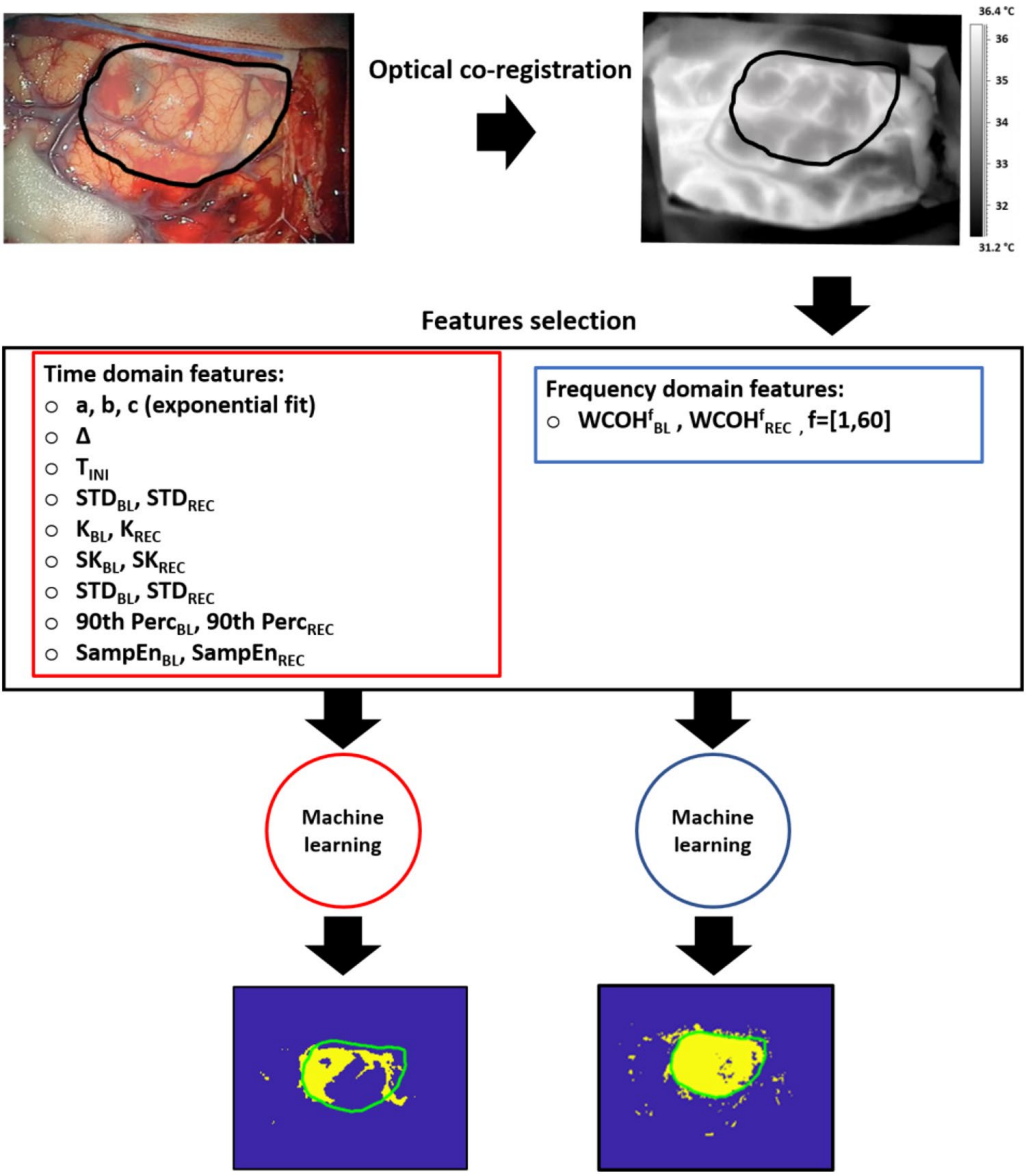
Pipeline of the processing approach developed in the present study. The optical co-registration between visible and thermal imaging is necessary to have an indication of the boundary of the cancer area on IRI. Then, TD and FD fetaures are extracted for the only BL and BL + REC phases. Last, supervised machine learning approaches are developed to classify healthy tissue from cancer areas, for each patient

To project the tumor area on the thermal imagery, a co-registration approach between the visible and thermal imagery was performed using the *Control Point Selection Tool* of Matlab 2021b. Corresponding couples of points between the two images of the exposed cortex were selected and then used to find the optimal affine geometrical transformation between the two images, thus allowing to transfer the boundary of the tumor region from visible imaging to IR imaging (Fig. 2a and b).

### Thermal features extraction

Thermal signals from each pixel were analyzed through both time domain (TD) and frequency domain (FD) approaches.

Concerning the TD analysis, the following features were computed:

- Coefficients of the exponential fit (a, b, c): the thermal signal associated to the recovery phase was modelled through an exponential fit, and the coefficients of the model were considered as indicative of the thermal behavior. Figure 1d shows a typical thermal signal behavior during the recovery after cold stress (highlighted in orange in the graphic). Specifically, the equation of the exponential fitting function is reported in Eq. 1:
  1 $$y=a\bullet \left(1-{e}^{bx}\right)+c$$
- where, *a* represents the difference between the temperature at the end and at the beginning of the recovery phase (i.e. the ideal asymptotic value after the thermal recovery), *b* is the inverse of the time constant (τ), and *c* is the initial value of the temperature, after the cold saline injection. For each pixel, the fit has been considered only if the goodness of fit (*R*) is higher than 0.8, otherwise the exponential fit has been discarded for the specific pixel.
- Temperature Variation (Δ): difference between the average value of the signal in the first 10 s and in the last 10 s of the whole experimental procedure.
- Initial Temperature (T_INI_): average value of the signal 30 s before the cold stress.
- Standard Deviation (STD_BL_, STDBL_REC_): standard deviation of the raw thermal signals evaluated in the baseline and recovery phases, respectively.
- Kurtosis (K_BL_, K_REC_): kurtosis of the raw thermal signals evaluated in the baseline and recovery phases, respectively.
- Skewness (SK_BL_, SK_REC_): skewness of the raw thermal signals evaluated in the baseline and recovery phases, respectively.
- 90th percentile (90th Perc_BL_, 90th Perc_REC_): 90th percentile of the raw thermal signals evaluated in the baseline and recovery phases, respectively.
- Sample Entropy in the baseline and recovery phases (SampEn_BL_, SampEn_REC_): it is defined as the negative natural logarithm of the conditional probability U that signal subseries of length m (pattern length) that match pointwise within a tolerance r (similarity factor) also match at the m + 1 point (Eq. 2) [30].
  2 $$\mathrm{SampEn}\left(\mathrm{m},\mathrm{r},\mathrm{N}\right)=-\mathrm{ln}\left[\frac{{\mathrm{U}}^{\mathrm{m}+1}(\mathrm{r})}{{\mathrm{U}}^{\mathrm{m}}(\mathrm{r})}\right]$$
- In this study, m=2 and r=0.2·SD (SD is the Standard Deviation of the signal) where chosen [31].

Concerning the frequency-domain (FD) analysis, the wavelet coherence (WCOH) between the average temperature time course of a randomized portion of pixels extracted from the tumor area and the temperature signals of each pixel of the thermal video was computed. WCOH is a measure of the correlation between two signals in the time–frequency plane. In this particular case, WCOH was considered for 60 frequency bands, in the range [0.015,2] Hz.

In detail, for each pixel a set of 60 values of WCOH were available, indicated as WCOH^f^_BL_ and WCOH^f^_REC_, evaluated in the baseline and recovery phases, respectively.

The average over-time of the amplitude of the WCOH for each frequency band was considered as indicative of the thermal functioning of each pixel.

Definitively, both the TD and FD analysis were performed considering only the baseline and the whole time course (BL + REC) (Fig. 2).

### TD and FD features analysis

**Fig. 3:**
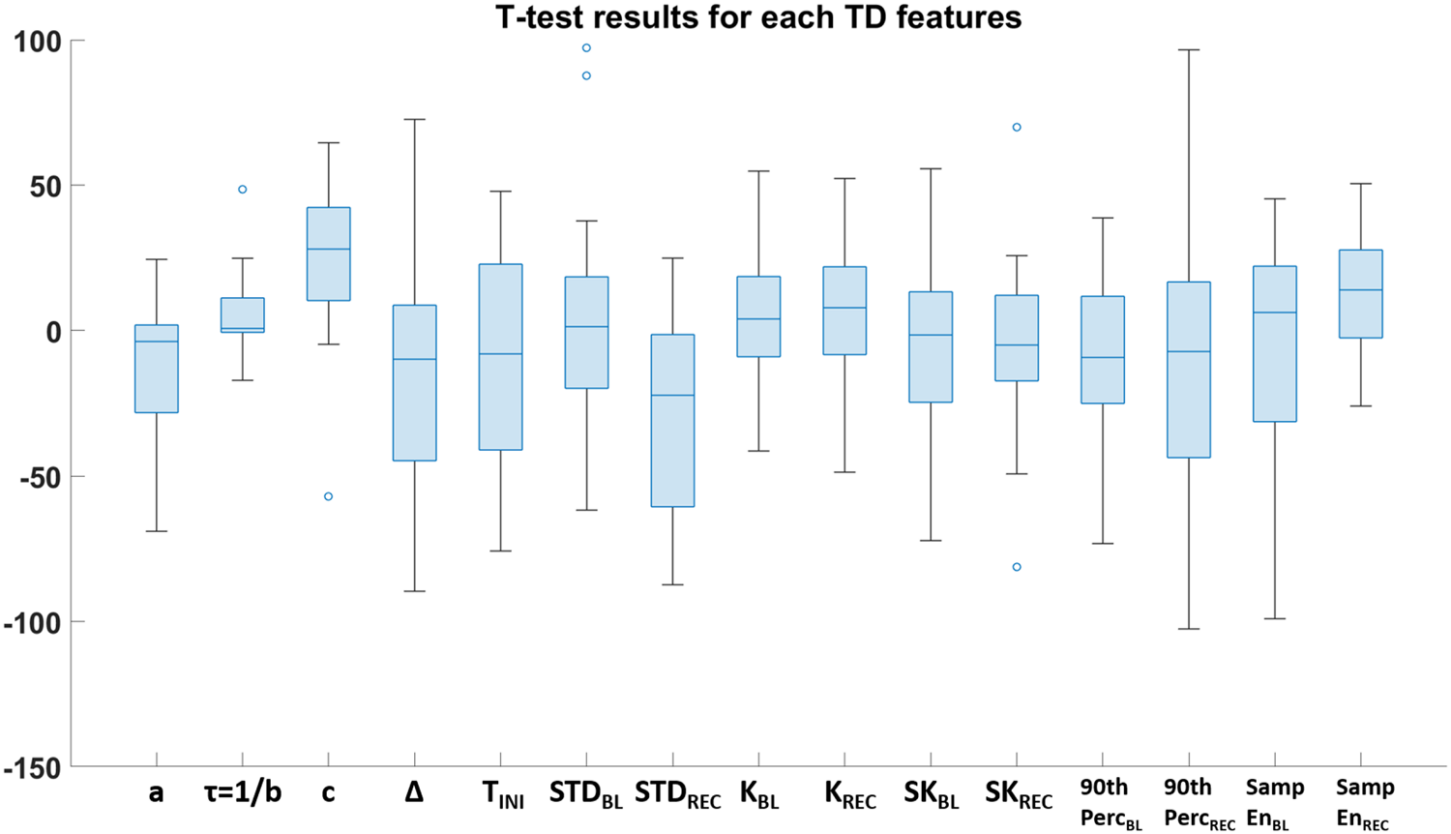
Whisker plot of t-values resulted from statistical t-tests for each TD feature. The comparison is between the features values relatively to class 0 vs. class 1 pixels

Preliminarily to the application of machine learning approaches, input features, being them TD- or FD- based features, were inspected and statistical t-test were applied to understand the underlying phenomena. T-test were performed for each features to test the significance of the comparison between class 0 (associated to healthy tissue) and class 1 (associated to the tumor pixels) relatively to each patient. The output was Bonferroni-corrected for multiple comparisons. Figure 3 shows the results for the t-test relative to each TD features.

The t-test comparisons were statistically significant for each feature except for some isolated cases. In particular, feature a was not significant for 2 subjects out of 13, feature b was not significant for 6 subjects out of 13, feature Δ was always significant except for 1 patient, STD_REC_ was not significant for 2 subjects out of 13 and SK_REC_ was not significant for 1 subjects out of 13.

**Fig. 4:**
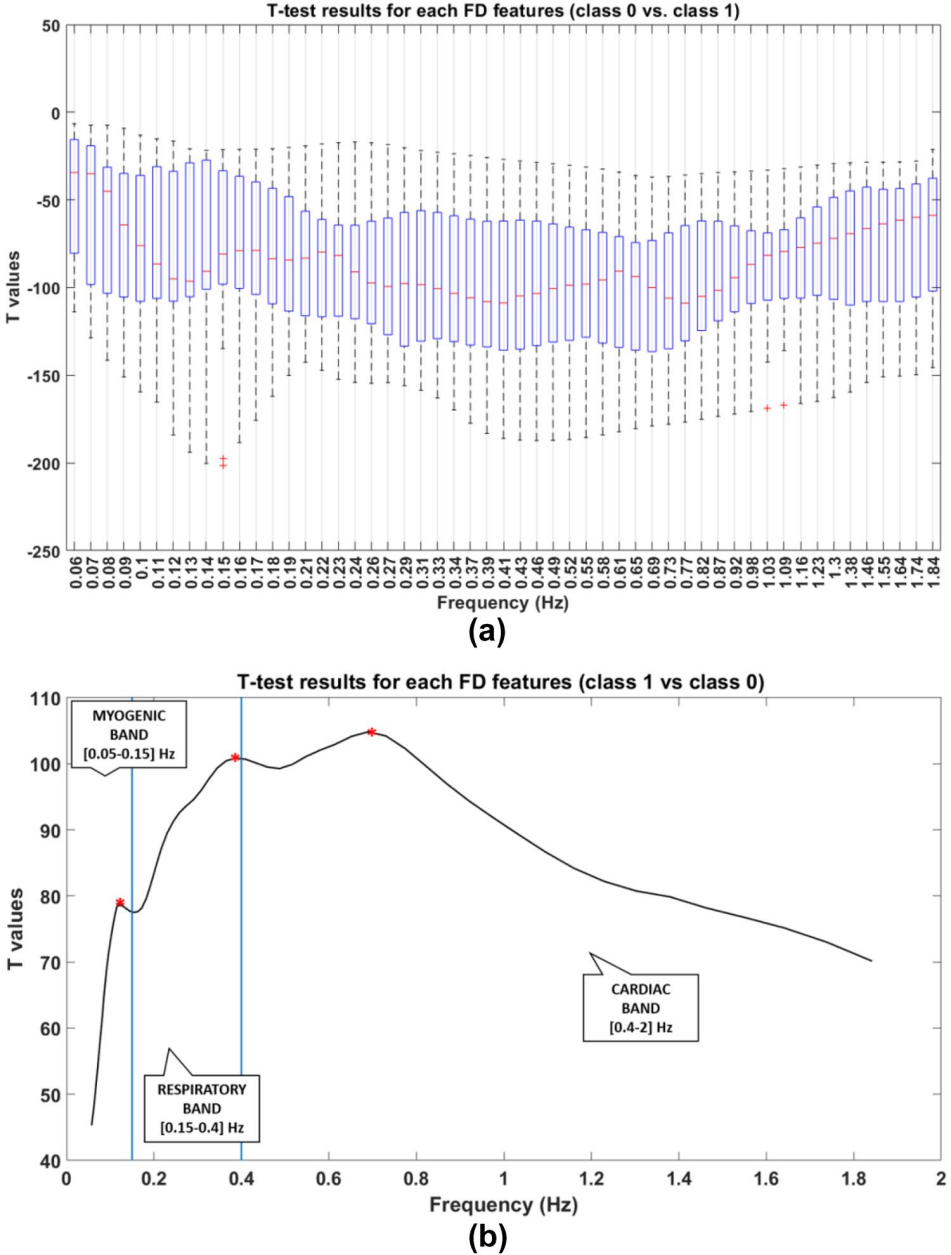
Representation of t-values resulted from statistical t-tests for each FD feature. **a** Whisker plot of t-values resulted from statistical t-tests for each FD feature. The comparison is between the features values relatively to class 0 vs. class 1 pixels. **b** Average of t-values among subjects. The comparison is between the features values relatively to class 1 vs. class 0 pixels, in order to have positive values. Maximum values are represented with red asterisks

Relatively to the FD features, t-test were performed to understand whether the 60 features could be representative of a discriminant behavior between healthy pixels (class 0) and tumor pixels (class 1). The output was Bonferroni-corrected for multiple comparisons. The t-test comparisons were statistically significant for each feature. Figure 4 reports the results of the t-test in two separated plots (Fig. 4a and b). In Fig. 4a a whisker plot of the t values relative to the frequency bands for the comparison class 0 vs. class 1 is shown. Figure 4b is, instead, relative to the contrast class 1 vs. class 0, to facilitate the interpretation of results, being represented by a positive amount of t-values. In this figure, the average of t-values among subjects are reported and maximum values of t are highlighted with red asterisks. Particularly the maximum value of t is obtained for f = [0.69–0.73] Hz in the Cardiac band ([0.4–2] Hz).

### Application of supervised machine learning

A Support Vector Machine (SVM) with radial basis function (RBF) kernel was employed to classify tumor pixels from healthy pixels [32]. Given the heterogeneity of the study sample, different models were developed for each participant. Particularly, for each patient, four different models were developed considering only the baseline or the entire time course relying on both time and frequency domain features (Fig. 2).

A subset of pixels randomly selected was used as a training set (20% of the pixels), another was used as a test set (20%) and the remaining pixels were used as a validation test. The tumor pixels were labeled as 1, whereas the healthy pixels were labeled as 0. For the training and test set, the classes were balanced, to avoid overfitting effect. To this aim, the larger class of the two was randomly down-sampled, to ensure the same class dimensionality. To test the generalization performances of the model, a k-fold cross-validation, with k = 10, was employed [33]. The cross-validation process ensures the generalizability of the models, allowing to estimate the performances of the classifiers.

## Results

**Fig. 5:**
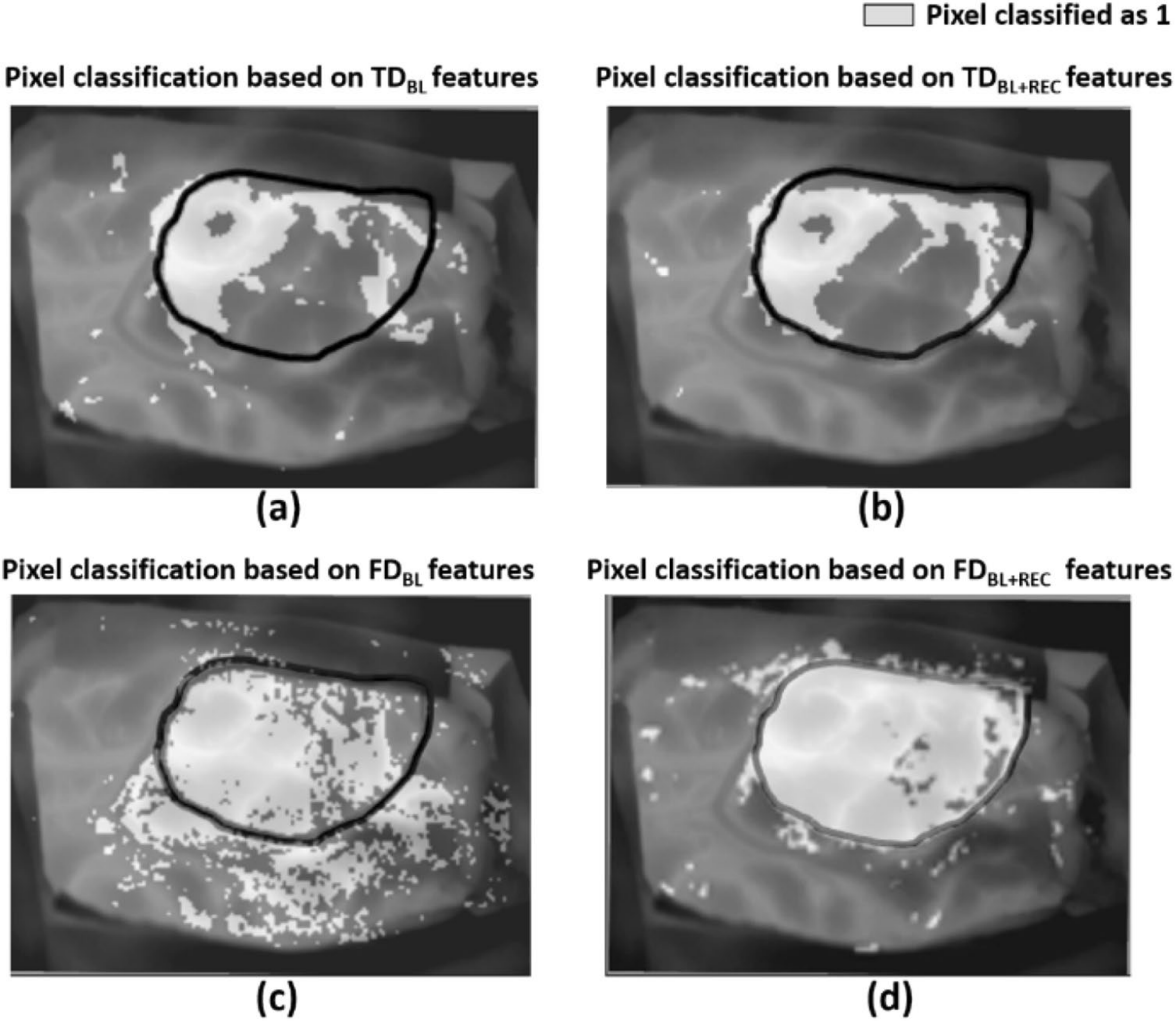
Outcome of classification models for an exemplificative subject relaying on: **a** TD features of the only BL; **b** TD features of the whole experiment (BL + REC); **c** FD features of the only BL; **d** FD features of the whole experiment (BL + REC). Black boundary is indicative of the tumor area whereas light grey pixels are the ones that the models classify as class 1 (i.e. tumor)

Figure 5 reports the results obtained for an indicative patient in segmenting the tumor area from thermal imaging.

Specifically, Fig. 5a shows the classification performance obtained with time domain features when considering only the baseline (TD_BL_), whereas Fig. 5b reports the classification obtained employing the time domain analysis for the entire time course (TD_BL+REC_). Figure 5c describes the segmentation reached using the frequency-domain features computed for the only baseline phase (TD_BL+REC_), whereas Fig. 5d shows the results obtained with the same approach computed during the whole experiment (TD_BL_).

**Fig. 6:**
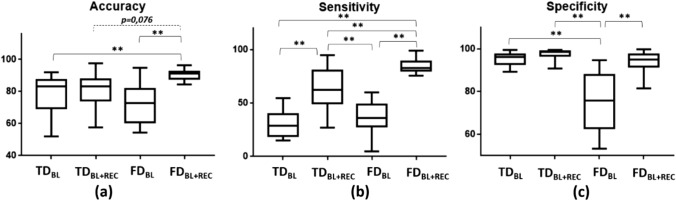
Average performances of the developed classifers: **a** average accuracy, **b** average sensitivity, **c** average specificity for the four categories of classifiers. Significant comparison are reported on the graphics (** = p <  < 0.01)

The performances obtained by the different models developed across all the participants are reported in Fig. 6. Particularly, the accuracy (Fig. 6a), the sensitivity (Fig. 6b) and the specificity (Fig. 6c) were considered to describe the performances of the model. For the sake of clarity, the mean values and standard deviation of these descriptors relative to the four categories of models are reported in Table 2.

**Table 2:** Mean values and standard deviations of the four categories of models relying on TD_BL_, TD_BL+REC_, FD_BL_, FD_BL+REC_ features

	Accuracy [%] (mean ± standard deviation)	Sensitivity [%] (mean ± standard deviation)	Specificity [%] (mean ± standard deviation)
TD_BL_ models	78.56 ± 12.82	30.87 ± 13.02	95.27 ± 2.97
TD_BL+REC_ models	79.86 ± 11.81	63.49 ± 20.96	97.45 ± 2.60
FD_BL_ models	72.30 ± 12.67	36.65 ± 14.42	75.45 ± 14.27
FD_BL+REC_ models	90.45 ± 3.32	84.64 ± 7.15	93.74 ± 5.00

A statistical comparison between these parameters was performed through a repeated measure ANOVA. Concerning the accuracy F(3,12) = 6.21, p <  < 0.01; multiple comparison revealed a statistical difference of FD_BL+REC_ with respect to all the other groups (Fig. 6a) with the exception of the comparison with TD _BL+REC_ for which there is a tendency towards significance (p = 0.076). With regard to sensitivity F(3,12) = 37.23, p <  < 0.01; multiple comparison analysis showed significant differences between all the groups except TD_BL_ vs FD_BL_ (Fig. 6b). Concerning the specificity F(3,12) = 21.87, p <  < 0.01; multiple comparison showed significant differences of FD_BL+REC_ with respect to all the other groups (Fig. 6c).

Furthermore, an analysis of the dependence of the performances of the FD_BL+REC_ models from the tumor category was performed to deep understand the relationship of the developed models to classify the different typologies of tumors.

**Fig. 7:**
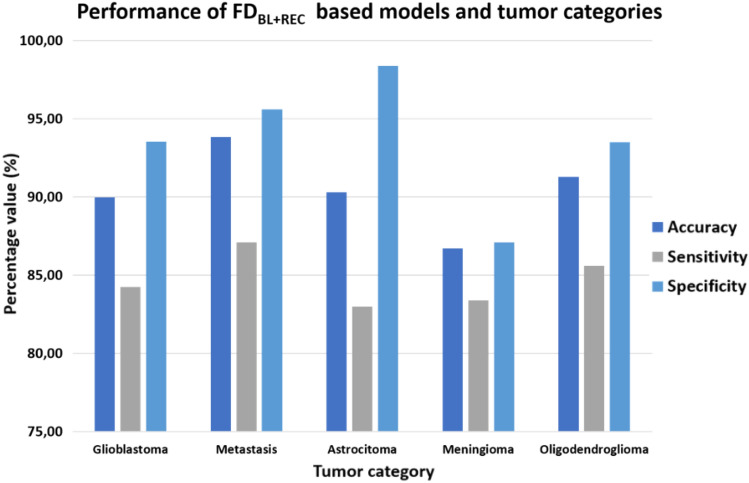
Bar plot of the performances indices of the FD_BL+REC_ models relatively to the tumor category

The present analysis was limited to the FD_BL+REC_ models which revealed to perform better with respect to the other models. Figure 7 represents the values of average accuracy, sensitivity, and specificity for the five categories of tumor of the sample dataset. Among all the tumor categories, FD_BL+REC_ models seemed to perform better for metastatic tumors, with the highest values of accuracy and sensitivity.

Finally, to compare the results of the developed approach with the state-of-the-art works in the field of thermal IR imaging, a summary of the performances of the available methods is reported in Table 3.

**Table 3:** Summary of the state-of-the-art works in the field of cancer detection using thermal infrared imaging

Authors	Field of application	Methodology	Performances
Wishart [15]	Breast cancer	Comparison of Infrared imaging detection with the gold standard technique (i.e. mammography)	For women < 50 years: sensitivity = 78% specificity = 75% For women aged 50–70 years: sensitivity = 72% specificity = 37%
Magalhaes [16]	Melanocytic nevi	Suppor vector machine (SVM)	Accuracy = 84.2% Sensitivity = 91.3%
Namdari [19]	Varicocele	- Statistical t-test and ANOVA - Comparison of the results with a gold standard methodology (i.e. Ultrasound method)	- Significant difference between the two groups of healthy samples and those with varicocele (p < 0.001) - Accuracy = 76%
Filippini [21]	Raynaud phenomenon	Deep convolutional neural network	Accuracy = 88% Sensitivity = 88% Specificity = 94%
Gorbach [24]	Humans’ brain cancer	ANOVA	Significant difference between the cortex overlying the tumor and the surrounding cortex (p < 0.01)
Papaioannou [25]	Rats’ brain cancer	ANOVA	Significant difference between the cortex overlying the tumor and the surrounding cortex (p < 0.05) and between the tumor and the core temperature (p < 0.05)
Kastek[28]	Humans’ brain and lung cancer	No statistical analysis reported	Observed temperature differences between the visualized surfaces of cancerous and healthy tissues

From the analysis of Table 3, it is possible to observe the lack of studies in the field of neurosurgery of methods relaying on machine learning approaches. The present works thus represents the first development of a machine-learning model, allowing also high accuracy, sensitivity and specificity in cancer detection and boundary identification of the lesion.

## Discussion

In this study, a non-invasive and contactless methodology, thermal infrared imaging (IRI), has been used to accurately detect the boundaries of the tumor tissue on the exposed cortex during neurosurgery. Thirteen subjects with heterogeneous tumors (Table 1) were enrolled. The experimental protocol consisted in a baseline (BL) phase, a cold stress phase, with cold physiological solution injection, and a recovery (REC) phase. Thermal imaging was acquired during the whole experiment.

After reporting the boundary location of the tumor lesion on thermal IR imaging by means of a co-registration with visible imaging, salient features were extracted in both time (TD) and frequency domain (FD) in the context of the only BL phase or relatively to the whole experiment (BL + REC). Different supervised machine learning based models were developed for each patient, given the heterogeneity of the tumors. The labels of the two classes (i.e., 0 for the healthy tissue pixels and 1 for the tumor tissue pixels) were given on the basis of the boundary defined by the neurosurgeon relying on pre-operative MRI.

A preliminary inspection of the features revealed statistical significance when comparing class 0 vs. class 1 pixels relatively to the values of both TD and FD features. In particular, referring to Fig. 3 the most influencing features in TD were the c parameter and the STD_REC_. The c parameter is related to the initial value of the temperature of the pixels after the cold saline injection and on average the t value is positive, meaning that, in general, the starting temperature after cold stress of the healthy pixels is higher than the tumor area pixels. This finding could be interpreted as the tendency of the tumor pixels not to react quickly to cold stress and to remain in the perturbed condition longer than the healthy pixels. The other most influencing parameter in TD features inspection was the standard deviation of the thermal signals of the pixels during REC phase, i.e. after the cold saline injection. The t-value, in this case, is negative meaning that the STD of class 1 pixels is higher than the STD of class 0 pixels during the thermal recovery. This result shows the difference of thermal characteristics of the two areas of the brain and reflects the scattered behavior of the tumor area with respect to the healthy regions, which behaves more uniformly.

Referring to the FD features (Fig. 4), instead, t-tests results showed statistical significance for all the analyzed frequency bands, and the maximum of the average t-value was at f = [0.69–0.73] Hz, which is in the Cardiac band (Fig. 4b). This finding means that the wavelet coherence is able to discriminate tumor from healthy pixels more efficiently in the above mentioned frequency band with respect to all the other bands under consideration. Referring to Fig. 4b, the results are represented for the contrast class 1 vs. class 0, therefore a high value of t means that the wavelet coherence in the tumor area pixels is higher compared to the healthy pixels. This means that the healthy pixels behave differently from the tumor area pixels for all the analyzed frequency bands, especially with a high impact on the cardiac band.

Concerning the developed supervised machine learning approach, the results showed the possibility to segment the tumor lesion with respect to the healthy brain regions with high performances with every one of the developed models, reporting an accuracy that on average is always more than 70% (Fig. 6a). Among the four models, the best in terms of accuracy was the FD based classifiers relaying on the whole experimental session features (BL + REC). In this case, the accuracy was on average 90.45%, whereas for FD based classifiers relaying on the only BL features it was 72.30%. With regard to TD based models the accuracies were 78.56% and 79.86% on average, for BL and BL + REC features respectively. Table 2 resumes the results of the developed classifiers.

Also, the sensitivity was higher for the FD classifiers relaying on the whole experimental session features (BL + REC), with 84.64% that was notably higher than the other models (30.87% for TD_BL_, 63.49% for TD_BL+REC_ and 36.65% for FD_BL_). The models with the highest specificity, instead, were the TD classifiers relaying on the whole experimental session features (BL + REC), with 97.45% on average that was similar to the levels of specificity of TD_BL_ and FD_BL+REC_ models, with 95.27% and 93.74%. The lowest specificity was reported for the FD_BL_ classifiers with a level of 75.45%.

The reported results demonstrated that FD classifiers relaying on the whole experimental session features (BL + REC) performed better with respect to the other three developed models, with high accuracy and sensitivity. This result can certainly be traced back to the fact that the input features are multiple and offer greater detail on the observed phenomenon. To note, referring to Fig. 6 and Table 2, it is possible to observe that also the FD classifiers relying on the only BL features had good performances and it is an important finding, because the classifier model would rely on a thermal imaging video of only 1 minute and without any additional measurement phase (i.e. cold stress), thus resulting more convenient during neurosurgical interventions.

In addition, an exploratory analysis was executed to relate the performances of the best models (FD_BL+REC_) to the tumor categories in the sample dataset. Among all the tumor categories, FD_BL+REC_ models seemed to perform better for metastatic tumors, with the highest values of accuracy and sensitivity. To note, the performances relative to the other classes of tumors were also very promising, being the values of accuracy, sensitivity and specificity always higher than 80%.

It is worth to note that the present work demonstrated that machine learning models based on FD features are more effective and performing that the TD features. This particular result can be traced back to the fact that the decomposition into frequency bands makes it possible to evaluate the characteristics of the signal, and in particular the specific correlation of the thermal signals in detailed frequency bands, with greater specificity and refinement with respect to the temporal signal analysis. This finding is of paramount importance also to understand the application of thermal IR imaging in the biomedical field. In fact, the IR imaging allows to assess the integration of several physiological mechanisms, which all together, affect the thermal pattern of a tissue (e.g., micro- and macro-circulation, metabolic activity of the tissue, exchange of heat with the environment) [34]. Frequency analysis of thermal signal permits of course to find the single most informative components of the underlying phenomena, allowing to obtain a more detailed insight on the dataset. Indeed, it has been largely employed in the field of thermal IR imaging applied on human studies [35–37]

It is of fundamental importance to observe that the present work is highly innovative given that it is the first time that a machine learning classifier relying on features extracted from a completely non-invasive and contactless technique has been used to segment the tumor area from the health tissue with outstanding performances. Table 3 shows a summary of the state-of-the-art works in the field of cancer detection and it is possible to note that the present model is the first machine-learning based approach ever developed in the IR imaging research context.

However, several limits affected the present study. The first is related to the limited sample size. Machine learning models are based on supervised learning and the performances are highly affected by the numerosity of the study sample. Increasing the numerosity of the patients could reduce the overfitting risk. Of note, the results are cross-validated, hence the generalization performances of the model are indeed investigated, but enlarging the sample size could improve the classification outcomes. Moreover, the effect of the limited sample size can be also observed in Table 1, which shows that for some patients the thermal behaviour is not always in line with that reported in the literature, especially for patients affected by glioblastoma. In this case, many patients showed higher average basal temperature of the tumor area compared to the healthy tissue. This result could be due to the inclusion of blood vessels in the region of interest of the tumor, thus increasing the average temperature of the area. However, this is beyond the scope of this work which focuses on identifying the boundaries of the tumor area to support the neurosurgeon in brain resection. Indeed, this allows to highlight the good qualities of the developed models to classify the nature of the pixels, focusing on the single frequency components, thus allowing to consider various physiological aspects of the underlying process. In addition, related to the limited sample size, it has not been possible to apply more sophisticated and modern approaches, such as deep learning methods. In fact, deep learning approaches rely on a huge number of samples and it has been demonstrated that the sample size highly impacts on the model accuracy [38, 39].

Second, the best classifiers are obtained for features relying on a time period of acquisition of nearly three minutes and on an experimental session consisting on injection of cold physiological solution. This time slot could be reduced to one minute at the price of decreasing performances. However, spraying with saline is a common practice during neurosurgery, thus constituting a mild limitation.

## Conclusions

The present work describes a novel method for the tumor segmentation of the exposed cortex during neurosurgery. Comparing different typologies of supervised machine learning methods based on time domain or frequency domain features, it has been possible to define the best category of classifiers relying on a non-invasive and contactless technique, the thermal infrared imaging. Model based on frequency domain features has revealed to be the best solution in terms of classification performance (Average Accuracy = 90.45%; Average Sensitivity = 84.64%; Average Specificity = 93,74%) with respect to the time domain features based model (Average Accuracy = 79.86%; Average Sensitivity = 63.49%; Average Specificity = 97.45%). An innovative tool is in this way now available for neurosurgeons, paving the way to new approaches for intra-operative assessment of tumor areas. Future perspective are in the direction of increasing the sample size, enrolling a more relevant number of patients, thus allowing to implement solutions based on deep learning methods, such as artificial neural networks. Relying on these models will allow and automatic and more accurate identification of the boundaries of the tumor lesion.

In conclusion, the most important contributions of the present work are highlighted below:

- A completely non-invasive, contactless, and portable technology (i.e., thermal IR imaging) has been employed to detect the tumor boundaries in an intraoperative context.
- Machine learning approaches has been developed on both time and frequency domain features, extracted from IR imaging.
- The method based on frequency features revealed to be the best machine learning solution in terms of performance.
- The model developed is an innovative solution relying on thermal IR imaging, allowing to identify with high accuracy the boundary of the tumor, at a pixel level.

## Data Availability

The data presented in this study are available on request from the corresponding author. The data are not publicly available due to privacy issues. The codes developed for the purpose of the study are available on request to the corresponding author.
